# Presence and Significant Determinants of Cognitive Impairment in a Large Sample of Patients with Multiple Sclerosis

**DOI:** 10.1371/journal.pone.0069820

**Published:** 2013-07-29

**Authors:** Martina Borghi, Marco Cavallo, Sara Carletto, Luca Ostacoli, Marco Zuffranieri, Rocco Luigi Picci, Francesco Scavelli, Harriet Johnston, Pier Maria Furlan, Antonio Bertolotto, Simona Malucchi

**Affiliations:** 1 Department of Mental Health, “San Luigi Gonzaga” Hospital Medical School, University of Turin, ASL TO3, Orbassano, Italy; 2 Department of Translational Medicine, “Amedeo Avogadro” University of Eastern Piedmont, Novara, Italy; 3 School of Psychology, University of St Andrews, St Andrews, Scotland, United Kingdom; 4 Neurologia 2 – CRESM (Regional Reference Centre for Multiple Sclerosis), “San Luigi Gonzaga” Hospital Medical School, Orbassano, Italy; Friedrich-Alexander University Erlangen, Germany

## Abstract

**Objectives:**

To investigate the presence and the nature of cognitive impairment in a large sample of patients with Multiple Sclerosis (MS), and to identify clinical and demographic determinants of cognitive impairment in MS.

**Methods:**

303 patients with MS and 279 healthy controls were administered the Brief Repeatable Battery of Neuropsychological tests (BRB-N); measures of pre-morbid verbal competence and neuropsychiatric measures were also administered.

**Results:**

Patients and healthy controls were matched for age, gender, education and pre-morbid verbal Intelligence Quotient. Patients presenting with cognitive impairment were 108/303 (35.6%). In the overall group of participants, the significant predictors of the most sensitive BRB-N scores were: presence of MS, age, education, and Vocabulary. The significant predictors when considering MS patients only were: course of MS, age, education, vocabulary, and depression. Using logistic regression analyses, significant determinants of the presence of cognitive impairment in relapsing-remitting MS patients were: duration of illness (OR = 1.053, 95% CI = 1.010–1.097, p = 0.015), Expanded Disability Status Scale score (OR = 1.247, 95% CI = 1.024–1.517, p = 0.028), and vocabulary (OR = 0.960, 95% CI = 0.936–0.984, p = 0.001), while in the smaller group of progressive MS patients these predictors did not play a significant role in determining the cognitive outcome.

**Conclusions:**

Our results corroborate the evidence about the presence and the nature of cognitive impairment in a large sample of patients with MS. Furthermore, our findings identify significant clinical and demographic determinants of cognitive impairment in a large sample of MS patients for the first time. Implications for further research and clinical practice were discussed.

## Introduction

Multiple Sclerosis (MS) is a chronic neurodegenerative disease typically characterized by white matter lesions, axonal damage and cerebral atrophy [1], [2]. MS affects both the neurological and the psychological domains. Most patients are diagnosed between 20 and 50 years of age, and women are affected two to three times as often as men [3]. In a study conducted in central Italy, the overall prevalence rate was 95 cases per 100,000. In keeping with previous results, a higher prevalence rate for females than males was recorded. Age-specific prevalence rate was higher in the 25–34 year, 35–44 year and 45–54 year age groups [4].

Cognitive impairment is a common clinical feature of MS, with current prevalence rates ranging from 30% to 70% [5], [6]. MS negatively affects various aspects of cognitive functioning including attention [7], information processing abilities [8], [9], processing speed [10], new learning [11] and memory [12]. It is relevant to note that cognitive impairment does not simply reflect problems in performing cognitive tasks, but is often associated with reduced functional status [13], [14]. In addition, cognitive impairment often has a deleterious impact on patients' occupational and social functioning, as well as on their overall quality of life [13], [15]. For example, it has been shown that people with MS who have cognitive impairment - as opposed to those with only the physical signs of the disease - were less likely to be employed, were engaged in fewer social and vocational activities, had greater difficulties in carrying out routine household tasks, and were more vulnerable to psychiatric illness [6]. Neuropsychological batteries have been developed to investigate cognitive impairment in MS. One of the most widely used cognitive batteries is the Brief Repeatable Battery of Neuropsychological tests (BRB-N; [16]), used for both research and clinical purposes. It encompasses tests tapping the cognitive functions affected by MS, and recent research have shown that some of these tests (e.g., the Selective Reminding Test, the Symbol Digit Modalities Test, and the Paced Auditory Serial Addition Test) have a higher sensitivity than others to detect deficits in this clinical population [17]. Thus, the need of cognitive assessment in patients with MS is gaining increasing attention in standard clinical routine.

To date there is no clear and robust evidence about what demographic and clinical variables may lead to an increased probability to developing cognitive impairment during the course of MS. To the best of our knowledge, previous studies have not specifically investigated the significant determinants of cognitive impairment in large samples of patients with MS. In the present study we recruited a large sample of patients with MS (N = 303) and a large sample of healthy controls (N = 279). All of the participants underwent the BRB-N with the twofold aim of investigating the presence and the nature of cognitive impairment in a large sample of MS patients, and identifying significant determinants of cognitive impairment in this clinical population.

## Methods

### Ethics Statement

Informed written consent was obtained from all of the participants. The study was granted approval by the Research Ethics Committee of the “San Luigi Gonzaga” Hospital Medical School of Orbassano (Italy), and was conducted in accordance with the Declaration of Helsinki.

### Participants

Three hundred and three patients with MS (91 males and 212 females) were consecutively recruited from May 2010 to June 2012 from the CReSM (Regional Reference Center for Multiple Sclerosis, affiliated with the University Hospital “San Luigi Gonzaga” of Orbassano, Italy), an Italian reference center for the diagnosis and treatment of patients with MS. All of the patients underwent detailed biological and clinical investigations, and received a definite diagnosis of MS, according to the standard International criteria [18], by neurologists expert in the diagnosis of MS (more than 10 years of clinical experience). Patients with possible MS or clinically isolated syndrome were not included in the study. In terms of MS status, 267 patients out of 303 (i.e. 88%) were classified as relapsing-remitting (RR), 9 (3%) as primary progressive (PP), 21 (7%) as secondary progressive (SP), and 6 (2%) as relapsing-progressive (RP). In total, 88% of the patients were classified as having a relapsing-remitting course of the disease, whereas 12% were classified as having a progressive course of the disease.

Inclusion criteria were as follows: definite diagnosis of MS according to the standard International criteria; more than 18 years old; fluent Italian speakers. Patients under high dosage of corticosteroids at the time of the recruitment were temporarily excluded, and they were administered the neuropsychological battery one month after the interruption of the drug treatment. Exclusion criteria were as follows: presence of severe psychiatric disorders such as psychosis or bipolar disorder; presence of severe medical conditions other than MS such as diabetes, stroke or traumatic brain injury; drug or alcohol abuse; suicide attempts; overt dementia; and serious eye disorders (such as diplopia).

Two hundred seventy nine healthy controls (84 males and 195 females) were recruited among the health professionals working at the University Hospital “San Luigi Gonzaga” of Orbassano (Italy) and among the caregivers of patients admitted to the various departments of the hospital. None of the healthy controls recruited were consanguineous of the patients with MS involved in the present study. Through a brief clinical interview based on the one reported by Green [19], it was established that none of the healthy controls recruited had a positive history of neurological or psychiatric disorders, of alcohol and drug abuse, or serious medical conditions.

### Procedure

The participants were administered the neuropsychological and neuropsychiatric measures detailed below at the University Hospital “San Luigi Gonzaga” of Orbassano (Italy) by expert clinical neuropsychologists (MB, SC, FS).

#### Neuropsychological measures

All of the participants were administered the Brief Repeatable Battery of Neuropsychological Tests (BRB-N) for Multiple Sclerosis [16] (Rao & the Cognitive Study Group of the National Multiple Sclerosis Society, 1990), a neuropsychological battery sensitive to the cognitive deficits that typically characterize MS. The BRB-N encompasses the following tests: the Selective Reminding Test (SRT), a test for verbal memory that provides measurement of learning and delayed recall capacity. It yields three different scores: the Selective Reminding Test-Long Term Storage (SRT-LTS), that provides a measure of the storage capacity in long-term memory; the Selective Reminding Test-Consistent Long Term Retrieval (SRT-CLTR), that provides a measure of the consistency of the recovery in long-term memory; the Selective Reminding Test-Delayed (SRT-D) a delayed recall of the words of the previously learned. The Spatial Recall Test (SPART), a test of learning and delayed recall of visuo-spatial items. It yields two scores: the SPART immediate recall score, and the SPART delayed recall score. The Symbol Digit Modalities Test (SMDT), a test of attention and of speed of information processing. The Paced Auditory Serial Addition Test (PASAT), that assesses the speed of information processing, the working memory, and the sustained attention. It encompasses two separate sub-tests (PASAT-2 and PASAT-3) in which the interval between two consecutive items changes (2 or 3 seconds, respectively). Finally, the Word List Generation (WLG), a semantic verbal fluency task.

To operationally define the construct of ‘cognitive impairment’, we used the criteria proposed by Amato et al. [20], a failure in at least two BRB-N tests, with scores at least 1.5 SD below the scores of healthy controls. Thus, if a patient had zero or one BRB-N test score at least 1.5 SD below that of healthy controls the patient was considered to have no cognitive impairment. If a patient had two or more BRB-N test scores at least 1.5 SD below that of healthy controls, the patient was considered to have cognitive impairment. In order to differentiate the degree of severity of deficits, if a patient had two BRB-N test scores below that of healthy controls , the patient was considered to have a mild degree of cognitive impairment. If a patient had three BRB-N test scores below that of healthy controls, the patient was considered to have moderate cognitive impairment. Finally, if a patient had four or more BRB-N test scores below that of healthy controls, the patient was considered to have severe cognitive impairment.

Furthermore, two additional cognitive measures were administered to all of the participants: the Brief Intelligence Test (TIB; [21]), functionally equivalent to the National Adult Reading Test [22]; and the sub-test Vocabulary of the Wechsler Adult Intelligence Scale [23]. Both of them are well established measures aiming at estimating the pre-morbid Intelligence Quotient (TIB), and the level of verbal intellectual functioning (Vocabulary).

#### Neuropsychiatric measures

The participants were administered the following two measures: the Hospital Anxiety and Depression Scale (HADS; [24]), a 14-item self-assessment scale that provides a valid and reliable measure of severity of anxiety and depression; and the Fatigue Severity Scale (FSS; [25]), a nine-item one-dimensional questionnaire assessing the severity of fatigue.

Lastly, patients with MS received a score from their neurologists on the Expanded Disability Status Scale (EDSS, [26]), to monitor their level of disability presented at the time of the current neuropsychological assessment.

### Statistical analyses

Parametric tests were used due to the large sample size and because the graphical exploration of the data by means of box plots and Q-Q plots indicated an acceptable distribution of the variables of interest. Statistical analyses were as follows: first, the comparison of the two groups (patients and healthy controls) on the demographic and clinical variables was performed via a series of t-tests. Second, in order to take into account the possible different profile of patients with different course of the MS [27], MS patients were divided into two sub-groups: patients with a relapsing-remitting course of MS (i.e. RR-MS), and patients with a progressive course of MS (i.e. prog-MS). These three groups (RR-MS, prog-MS, and healthy controls) were compared on the demographic and clinical variables via an ANOVA procedure, with Hochberg's GT2 post-hoc test. Third, participants' performances on the neuropsychological measures were compared via t-tests or ANOVAs, as appropriate. Finally, we performed multiple regression analyses in order to identify the significant predictors of the BRB-N test scores more sensitive to cognitive impairment in MS (SRT, SDMT, and PASAT-3), as well as simple and multiple logistic regression analyses in order to detect the influence of demographic and clinical variables on the presence of significant cognitive impairment in MS.

A *p* value<0.05 was accepted as statistically significant throughout all analyses. Statistical analyses were performed using SPSS© version 18.0 (Statistical Package for the Social Sciences).

## Results

First, we identified the presence and the degree of cognitive impairment in our sample of patients with MS according to the definition of cognitive impairment used by Amato et al. [20] previously reported. One-hundred ninety-five patients (195/303, 64.4%) did not show the presence of cognitive impairment. One-hundred and eight patients (108/303, 35.6%) presented with some degree of cognitive impairment at the time of testing, in keeping with previous studies [28]. Of those with cognitive impairment, 34/108 (31.5%) presented with a mild degree of cognitive impairment; 31/108 (28.7%) with a moderate cognitive impairment; 43/108 (39.8%) presented with a severe cognitive impairment.

### Patients with MS versus healthy controls

The two groups of participants were well matched for age (t_(580)_ = 1.863, NS), gender (χ^2^<0.001, NS), and years of formal education (t_(580)_ = 1.273, NS).

#### Neuropsychiatric measures

Regarding the HADS, five patients and one healthy control refused to complete the measure. One patient and five healthy controls did not complete the FSS at the time of the neuropsychological assessment. All of the other scores have been collected and used in the statistical analyses. As expected, the two groups of participants (patients with MS and healthy controls) differed in HADS-anxiety (t_(571)_ = 2.212, p<0.05) and HADS-depression (t_(571)_ = 4.503, p<0.01). In addition, the level of fatigue, FSS, was significantly different too (t_(574)_ = 10.395, p<0.01). This is in line with previous studies that repeatedly showed the presence of neuropsychiatric symptoms in patients with MS, as compared to healthy controls. Table 1 reports the demographic and neuropsychiatric variables of interest.

**Table 1:** Demographic and clinical variables of patients with MS and healthy controls.

Variable	MS patients mean (SD)	healthy controls mean (SD)	t-test (df) *or* χ^2^
	(n = 303)	(n = 279)	
*Participants' characteristics*			
Age in years	43.07 (10.79)	44.80 (11.70)	1.863 (580) NS
Gender (M∶F)	91∶212	84∶195	?^2^<0.001 NS
Education in years	12.76 (3.64)	13.16 (4.04)	1.273 (580) NS
Duration of illness in years	10.87 (7.26)	-	-
*Clinical measures*			
HADS–anxiety	6.74 (3.58)	6.09 (3.45)	2.212 (571)*
HADS–depression	5.84 (3.92)	4.50 (3.15)	4.503 (571)**
HADS–total	12.58 (6.75)	10.58 (5.93)	3.749 (571)**
FSS	36.23 (15.21)	24.36 (11.78)	10.395 (574)**
EDSS	2.43 (1.92)	-	-

* p<0.05;

** p<0.01;

df = degrees of freedom; EDSS = Expanded Disability Status Scale; FSS = Fatigue Severity Scale; HADS = Hospital Anxiety and Depression Scale; NS = Not Significant.

#### Neuropsychological measures

Regarding the neuropsychological measures, one patient did not complete the SRT-LTS and the SRT-CLTR scores. Twelve patients and six healthy controls did not complete the PASAT-3, and 28 patients and 10 healthy controls refused to do the PASAT-2, due to the high cognitive demand of these two tasks. Two patients did not perform the WLG and the subtest Vocabulary (WAIS), and one healthy control did not perform the TIB, due to time constraints. All of the other scores were collected and used in the statistical analyses. As expected, patients and healthy controls differed in all of the BRB neuropsychological tests. However, it is relevant to note that the two groups were well matched for pre-morbid IQ, as measured by the TIB (t_(579)_ = 1.041, NS). In addition, the number of errors on the TIB did not differ significantly between the two groups of participants (t_(579)_ = 0.738, NS). Table 2 reports the details of the performance on the neuropsychological tests.

**Table 2:** Neuropsychological measures in patients with MS and healthy controls.

Variable	MS patients mean (SD)	healthy controls mean (SD)	t-test (df)
	(n = 303)	(n = 279)	
WAIS–Voc	45.01 (12.12)	48.01 (12.37)	2.957 (578)**
TIB–IQ	111.70 (6.56)	112.27 (6.63)	1.041 (579) NS
TIB–errors	4.53 (5.32)	4.21 (5.09)	0.738 (579) NS
SRT–LTS	37.89 (14.46)	44.45 (13.13)	5.704 (579)**
SRT–CLTR	27.50 (14.42)	34.89 (14.70)	6.108 (579)**
SPART	18.41 (5.29)	19.90 (4.61)	3.624 (580)**
SDMT	46.41 (13.10)	51.72 (10.32)	5.401 (580)**
PASAT–3	38.02 (13.60)	40.97 (11.86)	2.731 (562)**
PASAT–2	27.17 (10.57)	30.75 (10.27)	4.013 (542)**
SRT–D	7.30 (2.51)	8.28 (2.24)	4.928 (580)**
SPART–D	6.43 (2.30)	6.94 (2.00)	2.860 (580)**
WLG	22.28 (5.72)	23.28 (5.51)	2.131 (578)*

* p<0.05;

** p<0.01;

df = degrees of freedom; NS = Not Significant; PASAT = Paced Auditory Serial Addition Test; SDMT = Symbol Digit Modalities Test; SPART = Spatial Recall Test; SPART–D = Spatial Recall Test-Delayed; SRT–CLTR = Selective Reminding Test-Consistent Long Term Retrieval; SRT–D = Selective Reminding Test-Delayed; SRT–LTS = Selective Reminding test-Long Term Storage; TIB–errors = Brief Intelligence Test-Errors; TIB–IQ = Brief Intelligence Test–Intelligence Quotient; WAIS–Voc = WAIS Vocabulary; WLG = Word List Generation.

### Patients with RR-MS versus patients with prog-MS versus healthy controls

#### Neuropsychiatric measures

The three groups of participants did not differ in the level of anxiety (F_(2,570)_ = 2.667, NS), whereas they differed in the levels of depression (F_(2,570)_ = 19.776, p<0.01), with both the RR-MS and the prog-MS patients groups presenting with a higher level of depression, as compared to healthy controls. In addition, the level of fatigue was significantly different (F_(2,573)_ = 55.417, p<0.01), with both RR-MS and prog-MS patients presenting with a higher level of fatigue compared to healthy controls. Table 3 reports the demographic and neuropsychiatric variables of interest. Hochberg's GT2 post-hoc test was used.

**Table 3:** Demographic and clinical variables of patients with RR–MS, progressive MS, and healthy controls.

Variable	RR-MS patients mean (SD)	prog-MS patients mean (SD)	healthy controls mean (SD)	F *or* t-test
	(n = 267)	(n = 36)	(n = 279)	
*Participants' characteristics*				
Age in years	41.65 (9.98)	53.59 (10.85)	44.80 (11.70)	20.905** ¶ § 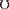
Gender (M∶F)	77∶190	14∶22	84∶195	-
Education in years	12.96 (3.64)	11.25 (3.37)	13.16 (4.04)	4.000* ¶ § 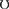
Duration of illness in years	10.31 (7.04)	15.06 (7.61)	-	3.760**
*Clinical measures*				
HADS–anxiety	6.69 (3.53)	7.12 (3.96)	6.09 (3.45)	2.667 NS
HADS–depression	5.52 (3.67)	8.29 (4.89)	4.50 (3.15)	19.776** ¶ § 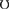
HADS–total	12.21 (6.53)	15.41 (7.79)	10.58 (5.93)	0.731 NS
FSS	35.77 (14.96)	39.61 (16.80)	24.36 (11.78)	55.417** § 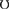
EDSS	2.04 (1.59)	5.29 (1.68)	-	11.393**

* p<0.05;

** p<0.01;

EDSS = Expanded Disability Status Scale; FSS = Fatigue Severity Scale; HADS = Hospital Anxiety and Depression Scale; NS = Not Significant; RR–MS = Relapsing-remitting Multiple Sclerosis; prog–MS = Progressive Multiple Sclerosis;

¶ prog–MS significantly different from RR–MS;

§ prog–MS significantly different from healthy controls;

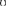
RR–MS significantly different from healthy controls.

#### Neuropsychological measures

In keeping with previous studies, prog-MS patients showed a more pronounced pattern of neuropsychological deficits, as compared to RR-MS patients. In all of the BRB-N measures, prog-MS patients had significantly lower scores than healthy controls, whereas RR-MS had lower scores than healthy controls' in most measures. In addition, in all of the measures (with the exception of the SPART score), prog-MS patients had significantly lower scores than RR-MS patients. In summary, prog-MS patients had the lowest scores and significantly differed from both RR-MS patients and healthy controls on the SRT-LTS, SRT-CLTR, SDMT, PASAT-3, PASAT-2, SRT-D, SPART-D, and WLG tests. In other words, the scores of the three groups of participants for each BRB measure laid on a ‘continuum’, ranging from prog-MS patients' score (the ‘lowest’ score) through RR-MS patients' score (the ‘intermediate’ score) to healthy controls' score (the ‘highest’ score). It is relevant to note that the three groups did not differ in terms of number of errors on the TIB (F_(2,578)_ = 2.590, NS), whereas the prog-MS patients differ in terms of TIB pre-morbid IQ (F_(2,578)_ = 3.881, p<0.05) and WAIS Vocabulary (F_(2,577)_ = 12.068, p<0.01) from the other two groups. Interestingly, RR-SM patients and healthy controls did not differ from one another on TIB pre-morbid IQ and WAIS Vocabulary. Table 4 reports the details of the performance of the three groups on the neuropsychological tests. Hochberg's GT2 post-hoc test was used.

**Table 4:** Neuropsychological measures in patients with RR–MS, progressive MS, and healthy controls.

Variable	RR-MS patients mean (SD)	prog-MS patients mean (SD)	healthy controls mean (SD)	F
	(n = 267)	(n = 36)	(n = 279)	
WAIS–Voc	46.01 (11.46)	37.64 (14.33)	48.01 (12.37)	12.068** ¶ §
TIB–IQ	112.06 (6.32)	109.05 (7.76)	112.27 (6.63)	3.881* ¶ §
TIB–errors	4.29 (5.06)	6.28 (6.81)	4.21 (5.09)	2.509 NS
SRT–LTS	39.68 (13.66)	24.24 (13.20)	44.45 (13.13)	38.006** ¶ § 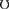
SRT–CLTR	29.10 (14.10)	15.35 (10.66)	34.89 (14.70)	34.019** ¶ § 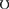
SPART	18.66 (5.38)	16.62 (4.22)	19.90 (4.61)	9.284** § 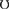
SDMT	47.93 (12.27)	35.10 (13.66)	51.72 (10.32)	35.414** ¶ § 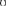
PASAT–3	38.98 (13.05)	30.78 (15.60)	40.97 (11.86)	10.107** ¶ §
PASAT–2	27.94 (10.28)	21.03 (10.94)	30.75 (10.27)	14.406** ¶ § 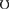
SRT–D	7.60 (2.38)	5.09 (2.37)	8.28 (2.24)	31.546** ¶ § 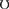
SPART–D	6.54 (2.32)	5.61 (1.95)	6.94 (2.00)	7.022** ¶ §
WLG	22.72 (5.61)	18.95 (5.49)	23.28 (5.51)	9.455** ¶ §

* *p*<0.05;

** *p*<0.01;

NS = Not Significant; PASAT = Paced Auditory Serial Addition Test; SDMT = Symbol Digit Modalities Test; SPART = Spatial Recall Test; SPART–D = Spatial Recall Test-Delayed; SRT–CLTR = Selective Reminding Test-Consistent Long Term Retrieval; SRT–D = Selective Reminding Test–Delayed; SRT–LTS = Selective Reminding test–Long Term Storage; TIB–errors = Brief Intelligence Test–Errors; TIB–IQ = Brief Intelligence Test–Intelligence Quotient; WAIS–Voc = WAIS Vocabulary; WLG = Word List Generation;

¶ prog–MS significantly different from RR–MS;

§ prog–MS significantly different from healthy controls;

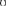
RR–MS significantly different from healthy controls.

### Predictors of SRT, SDMT, and PASAT-3 scores

According to Portaccio et al. [17], three tests of the BRB-N (SRT, SDMT and PASAT-3) are very sensitive in detecting cognitive impairment in MS (sensitivity of 94%, specificity of 84%, and accuracy of 89%). Thus, we focused on the significant predictors of these tests scores by considering firstly all of the participants in our analyses, and secondly only the patients with MS. The dichotomous variable “gender” was codified as 0 for males and 1 for females. The categorical variable “group” encompassed three distinct modalities (i.e. healthy controls, RR-MS patients, and prog-MS patients) and then could not be entered directly into the statistical models. Thus, we coded healthy controls as 0, RR-MS patients as 1, and prog-MS patients as 2. Healthy controls were used as a baseline, and we performed dummy coding to get two independent dichotomous variables (i.e. “relapse” and “prog”) to represent RR-MS patients and prog-MS patients in multivariate statistical models (backward procedure).

Regarding the SRT-LTS score, the significant predictors were “prog” (β = −0.245, p<0.001), “relapse”(β = −0.170, p<0.001), “age”(β = −0.289, p<0.001), “WAIS-Voc”(β = 0.239, p<0.001), “education” (β = −0.260, p<0.001), “gender” (β = 0.100, p = 0.007), and “FSS” (β = −0.119, p = 0.009). The statistical model encompassing these significant predictors yielded an Adjusted R^2^ = 0.246, leading to a 24.60% of variance explained.

Regarding the SRT-CLTR score, the significant predictors were “prog” (β = −0.237, p<0.001), “relapse”(β = −0.225, p<0.001), “age”(β = −0.300, p<0.001), “WAIS-Voc” (β = 0.292, p<0.001), and “education” (β = −0.258, p<0.001. The statistical model encompassing these significant predictors yielded an Adjusted R^2^ = 0.217, leading to a 21.70% of variance explained.

Regarding the SRT-D score, the significant predictors were “prog” (β = −0.206, p<0.001), “relapse”(β = −0.131, p = 0.02), “age”(β = −0.304, p<0.001), “WAIS-Voc” (β = 0.288, p<0.001), “education” (β = −0.233, p<0.001), “FSS”(β = −0.139, p = 0.003), and “HADS anxiety ” (β = 0.109, p = 0.011). The statistical model encompassing these significant predictors yielded an Adjusted R^2^ = 0.226, leading to a 22.60% of variance explained.

Regarding the SDMT score, the significant predictors were “prog” (β = −0.199, p<0.001), “relapse”(β = −0.170, p<0.001), “age”(β = −0.416, p<0.001), “WAIS-Voc” (β = 0.154, p = 0.003), “education” (β = −0.322, p<0.001), “TIB-errors” (β = −0.250, p<0.001), and “FSS”(β = −0.124, p = 0.001). The statistical model encompassing these significant predictors yielded an Adjusted R^2^ = 0.331, leading to a 33.10% of variance explained.

Finally, regarding the PASAT-3 score, the significant predictors were “prog” (β = −0.159, p<0.001), “relapse”(β = −0.107, p = 0.006), “age”(β = −0.146, p<0.001), “WAIS-Voc” (β = 0.221, p<0.001), “education” (β = −0.484, p<0.001), “gender” (β = −0.183, p<0.001), and “TIB-errors” (β = −0.252, p<0.001). The statistical model encompassing these significant predictors yielded an Adjusted R^2^ = 0.234, leading to a 23.40% of variance explained.

It is relevant to note that we repeated all of the previous analyses considering the group of MS patients only. The variable “course of the disease” codified RR-MS patients as 1, and prog-MS patients as 2. The results were as follows: regarding the SRT-LTS score, the significant predictors were “course of the disease” (β = −0.176, p = 0.001), “age”(β = −0.291, p<0.001), “education” (β = −0.230, p<0.001), “WAIS-Voc” (β = 0.319, p<0.001), and “HADS depression” (β = −0.159, p = 0.003). The statistical model encompassing these significant predictors yielded an Adjusted R^2^ = 0.271, leading to a 27.10% of variance explained.

Regarding the SRT-CLTR score, the significant predictors were “course of the disease” (β = −0.135, p = 0.016), “age” (β = −0.316, p<0.001), “education” (β = −0.265, p<0.001), “WAIS-Voc” (β = 0.295, p<0.001), and “HADS depression” (β = −0.199, p = 0.004). The statistical model encompassing these significant predictors yielded an Adjusted R^2^ = 0.246, leading to a 24.60% of variance explained.

Regarding the SRT-D score, the significant predictors were “course of the disease” (β = −0.144, p = 0.010), “age” (β = −0.278, p<0.001), “education” (β = −0.246, p<0.001), “WAIS-Voc” (β = 0.310, p<0.001), “HADS anxiety” (β = 0.186, p = 0.004), and “HADS depression” (β = −0.258, p<0.001). The statistical model encompassing these significant predictors yielded an Adjusted R^2^ = 0.260, leading to a 26.00% of variance explained.

Regarding the SDMT score, the significant predictors were “course of the disease” (β = −0.107, p = 0.041), “age” (β = −0.432, p<0.001), “education” (β = −0.400, p<0.001), “WAIS-Voc” (β = 0.179, p = 0.008), “TIB-errors” (β = −0.219, p = 0.001), and “HADS depression” (β = −0.155, p = 0.002). The statistical model encompassing these significant predictors yielded an Adjusted R^2^ = 0.335, leading to a 33.50% of variance explained.

Finally, regarding the PASAT-3 score, the significant predictors were “course of the disease” (β = −0.116, p = 0.042), “age” (β = −0.232, p<0.001), “education” (β = −0.507, p<0.001), “gender” (β = −0.163, p = 0.003), “WAIS-Voc” (β = 0.172, p = 0.019), and “TIB errors” (β = −0.245, p<0.001). The statistical model encompassing these significant predictors yielded an Adjusted R^2^ = 0.234, leading to a 23.40% of variance explained.

### Determinants of the presence of cognitive impairment in MS

In order to investigate which demographic and clinical factors may play a significant role in determining the presence or not of cognitive impairment in patients with MS, we performed simple and multiple logistic regression analyses with the presence of significant cognitive impairment in the group of MS patients as the dependent variable (YES/NO). As the size of the two groups of patients (RR-MS and prog-MS) was different, we performed two separate analyses for the two groups. We considered as presence of cognitive impairment (YES) having two or more BRB-N tests with scores at least 1.5 SD below the scores of healthy controls, whereas we considered as absence of cognitive impairment (NO) having zero or one BRB-N tests with scores at least 1.5 SD below the scores of healthy controls. We considered as potential predictors the following factors: “gender”, “education”, “age”, “duration of illness”, “EDSS”, “HADS-anxiety”, “HADS-depression”, “FSS”, “WAIS-Voc”, “TIB-IQ”, and “TIB-errors”. Regarding the RR-MS group, the variables “gender”, “education”, “HADS-anxiety” and “TIB-IQ” did not contribute significantly to the presence/absence of cognitive impairment in our patients. As expected, the predictors considered showed a significant degree of correlation. Firstly, because there is a substantive and meaningful relationship between factors such as years of illness and EDSS score (i.e. as the disease progresses, the degree of disability increases). Secondly, because the large sample size increases the likelihood of getting significant results. However, it is important to note that the significant correlations between predictors were low or moderate in size, and that each predictor still made a significant individual contribution to the cognitive outcome. Thus, the significant predictors were combined in a unique statistical model, leading to the final model encompassing three significant predictors: “duration of illness”, “EDSS”, and “WAIS-Voc”. Lastly, regarding the prog-MS group, the predictors considered in the models did not contribute significantly to the presence/absence of cognitive impairment. Tables 5 and 6 report the details of interest for the RR-MS patients and for the progressive MS patients, respectively.

**Table 5:** Logistic Regression (cognitive deficits YES/NO in RR–MS patients).

Predictor	*p* value	OR (95% CI) coefficient
*Simple Regression Analysis*		
Age in years	0.040*	1.028 (1.001–1.056)
Duration of illness in years	0.001*	1.068 (1.029–1.109)
EDSS	<0.001**	1.421 (1.201–1.680)
HADS-depression	0.002*	1.119 (1.041–1.204)
FSS	0.001*	1.032 (1.013–1.052)
WAIS-Voc	0.003*	0.964 (0.942–0.987)
TIB-errors	0.009*	1.069 (1.017–1.124)
*Multiple Regression Analysis*		
Duration of illness in years	0.015*	1.053 (1.010–1.097)
EDSS	0.028*	1.247 (1.024–1.517)
WAIS-Voc	0.001*	0.960 (0.936–0.984)

* *p*<0.05;

** *p*<0.001;

EDSS = Expanded Disability Status Scale; FSS = Fatigue Severity Scale; HADS = Hospital Anxiety and Depression Scale; TIB–errors = Brief Intelligence Test–Errors; TIB–IQ = Brief Intelligence Test–Intelligence Quotient; WAIS–Voc = WAIS Vocabulary.

**Table 6:** Logistic Regression (cognitive deficits YES/NO in prog–MS patients).

Predictor	*p* value	OR (95% CI) coefficient
*Simple Regression Analysis*		
Age in years	NS	1.025 (0.945–1.111)
Duration of illness in years	NS	1.024 (0.914–1.148)
EDSS	NS	1.253 (0.762–2.061)
HADS-depression	NS	1.076 (0.897–1.292)
FSS	NS	1.021 (0.971–1.073)
WAIS-Voc	NS	0.976 (0.920–1.036)
TIB-errors	NS	1.194 (0.941–1.515)

EDSS = Expanded Disability Status Scale; FSS = Fatigue Severity Scale; HADS = Hospital Anxiety and Depression Scale; NS = Not Significant; TIB–errors = Brief Intelligence Test–Errors; TIB–IQ = Brief Intelligence Test–Intelligence Quotient; WAIS–Voc = WAIS Vocabulary.

## Discussion

Cognitive impairment is a well-established clinical marker of MS. Several studies have shown that a significant proportion of patients with MS suffer from some degree of cognitive impairment [6], [20], leading to the conclusion that cognitive assessment should be part of the routine clinical assessment of these patients. However, to date most published studies do not involve large samples of patients, and specific investigation of the clinical and demographic determinants of cognitive impairment is lacking. In the present study, we recruited large samples of patients and healthy controls who underwent a neuropsychological assessment by means of a well-established neuropsychological battery (BRB-N). In addition, we investigated which variables influence participants' performance on the neuropsychological measures most sensitive to detect cognitive deficits in MS, and identified significant determinants of the presence of cognitive impairment in MS.

In our study, ‘cognitive impairment’ has been defined operatively as having two or more BRB tests with scores at least 1.5 standard deviations (SD) below the scores of healthy controls. Although there is no universal agreement on the most appropriate criterion to define operatively ‘cognitive impairment’ in MS, we followed this proposal as it has been widely used in previous studies [20], [29], and as it represents a good compromise between being too stringent and too lax in detecting cognitive impairment in MS. In keeping with previous studies, a significant proportion of MS patients (i.e. 35.6%) presented with some degree of cognitive impairment. As previous literature suggests that the different course of the disease (RR versus progressive) leads to different clinical profiles, we initially compared two groups of participants (MS patients and HC), and then we repeated all of the analyses after dividing the group of patients in two sub-groups, according to the course of the disease. For further analyses, the variable “course of the disease” was incorporated in all the statistical models examined.

The two groups of participants (MS patients and HC) were well matched for age, education, and gender. Patients presented with higher levels of anxiety and depression, as measured by the HADS. However, it is relevant to note that on average the levels of both anxiety and depression in the two groups were well below the clinical borderline range of values (i.e. scores between 8 and 12), allowing us to exclude on clinical grounds the presence of significant levels of anxiety or depression in our samples. Patients and HC still differed in terms of fatigue as measured by the FSS, with patients presenting higher levels of fatigue than controls. Regarding their neuropsychological profile, patients had significantly lower scores than HC on all of the measures administered, with the exception of the TIB-IQ, a reliable estimation of verbal pre-morbid IQ.

When the three groups of participants (RR-MS patients, prog-MS patients, and HC) were compared, patients with the progressive forms of the disease were characterized by older age, higher levels of depression and fatigue, as compared to RR-MS and HC, and higher level of disability than RR-MS. Regarding their neuropsychological profile, patients with the progressive form of the disease had significantly lower scores as compared to both RR-MS and HC in the vast majority of measures, whereas only in one measure (SPART) the two sub-groups of patients did not differ from each other.

These results confirm that patients presenting with the progressive forms of MS tend to present a more severe clinical and cognitive profile, as compared to patients with the relapsing-remitting form of the disease. Therefore we included this variable in our statistical models in subsequent multiple regression analyses to account for the significant role played by the different types of MS in predicting the presence of cognitive impairment.

As previous research identified some BRB-N sub-tests (i.e. SRT, SDMT, PASAT-3) as the most sensitive ones to detect specific cognitive impairment in MS [17], we investigated the significant predictors of these tests scores in all of the participants, and in MS patients only. In the overall group of participants, a significant proportion of the SRT-LTS score was explained by the course of the disease (progressive and relapsing-remitting), the age of patients, their level of education, their gender, their verbal competence (as estimated by the WAIS Vocabulary subtest), and their level of fatigue. A significant proportion of the SRT-CLTR score was explained by the course of the disease, the age of patients, their level of education, and their verbal competence. Lastly, a significant proportion of the SRT-D score was explained by the course of the disease, the age of patients, their level of education, their verbal competence, and their levels of fatigue and anxiety. As the SRT is a test of verbal memory that provides measurement of learning and delayed recall capacities, it was expected that younger age, higher level of intelligence, and verbal competence would be directly associated to higher scores on this task. As expected, being an MS patient was related to lower scores on these tasks, with progressive MS patients' performance more compromised than RR-MS patients' performance. However, we did not expect to identify an inverse relationship between level of education and the test scores.

Regarding the SDMT, a significant proportion of its score (adjusted R^2^ = 0.331) was explained by the variables course of the disease (progressive or relapsing-remitting), age, education, WAIS Vocabulary, TIB errors, and FSS score. As this task is a measure of attention and of speed of information processing, it was expected that younger age, higher level of verbal intelligence, and lower level of fatigue would be associated with higher scores. Being an MS patient was related to lower scores on these tasks, with progressive MS patients' performance being more compromised than RR-MS patients' performance. Again, we did not expect to detect a significant inverse relationship between level of formal education and the test score.

Lastly, a significant proportion of the PASAT-3 score (adjusted R^2^ = 0.234) was explained by the variables course of the disease, age, gender, education, WAIS Vocabulary, and TIB errors. As this task measures the speed of information processing, working memory functions and sustained attention, and as it is one of the most demanding task of the BRB-N, we expected there would be a significant role played by various demographic variables (age, gender) and pre-morbid cognitive measures (WAIS Vocabulary, TIB) in determining this score. Again, being an MS patient was related to lower scores on these tasks, with progressive MS patients' performance being more compromised than RR-MS patients' performance. A significant inverse relationship between the level of formal education and the test score was detected again.

An open issue remains why our results showed the presence of a significant inverse relationship between the level of formal education and these test scores. In fact, intuitively one would expect that higher levels of education should facilitate performance on these tasks, but in our pattern of results this was not the case. It is reasonable to assume that there is not a simple and linear explanation for this finding. Cognitive deficits are probably related to the nature and degree of white matter integrity, gray matter volume, and neural lesions. Thus, it is reasonable to expect that the degree of neural pathology may actually mediate the relationship between education and cognitive impairment, that cannot be meaningfully interpreted per se. However, further research should specifically investigate the inverse relationship between level of education and these tests scores, in order to foster our understanding of this link.

Another interesting point is that when we repeated all of the statistical analyses by considering MS patients only, the significant role of fatigue in contributing to the explanation of the cognitive scores of interest disappeared, while a significant role of depression appeared. A possible explanation for the first issue is that patients with MS are characterized by a higher level of fatigue than healthy controls. When controls were removed from the statistical analyses the level of fatigue no longer played a significant role in partially influencing cognitive scores. In other words, when considering MS patients only, fatigue played the role of a ‘constant’ rather than that of a significant ‘variable’. More controversial is the second issue. The level of depressive symptomatology (as measured by HADS depression) played a significant role in partially influencing the scores of the SRT and of the SDMT only in the group of MS patients. As these cognitive tasks are very sensitive to cognitive deficits in MS and are challenging, it is reasonable to assume that the presence of depressive symptoms in addition to the neurodegenerative disease can interfere with a satisfactory performance on these tasks that require an active and persistent involvement of the subject in terms of both cognitive and motor performance. However, further research should specifically investigate these two issues more deeply.

Lastly, we were interested in investigating the contribution of demographic and clinical factors to the presence (or absence) of cognitive impairment in our patients. As the size of the two groups of patients (RR-MS and prog-MS) was different, we decided to perform two separate analyses for the two groups. Regarding the RR-MS group, simple logistic regression analyses allowed us to identify seven variables (age, duration of illness, EDSS, depression, FSS, WAIS Vocabulary, and TIB errors) as significant individual predictors of the presence of cognitive impairment. Furthermore, multiple regression analyses combining these significant predictors allowed us to identify the duration of illness, the EDSS and the WAIS Vocabulary as significantly related to the presence of cognitive impairment. Thus, our results showed that the presence of cognitive impairment in RR-MS cannot be estimated simply by considering a single demographic or clinical factor. Conversely, estimating the possible presence of significant cognitive deficits requires us to take multiple demographic and clinical factors into account such as the duration of the disease, patient's disability status, and his/her pre-morbid level of verbal competence. Altogether, the combination of these factors yielded a statistical model that, while being parsimonious (i.e. it included only three of the 11 variables considered), presented an acceptable goodness-of-fit. Thus, this model can be used as a quick ‘screening tool’ to suggest the possible presence of cognitive impairment in RR-MS, that obviously will have to be confirmed via the administration of sensitive neuropsychological batteries. Regarding the prog-MS group, the predictors considered here did not contribute significantly to the presence/absence of cognitive impairment. So, the present findings do not allow us to identify significant predictors of cognitive impairment in this group of patients. However, it is reasonable to expect that with a larger sample size it would be possible to detect significant predictors also in progressive MS. Thus, further research is needed to investigate this important issue.

The present study presents some strengths. First, the large sample size allowed us to obtain reliable data about the presence, nature and significant determinants of cognitive impairment in MS. Second, only a few participants did not perform all of the neuropsychological tests or neuropsychiatric measures used. As we collected all of the data for the vast majority of participants, we are confident that our results are representative of the entire samples recruited and not limited to a portion of them. Finally, considering not only MS patients as a whole but also splitting the MS patients into two groups according to the type of MS (i.e. RR-MS, and prog-MS) allowed us to control for the very relevant clinical factor represented by the course of the disease, that should be taken into account also when dealing with the assessment of cognitive deficits in MS.

The study also presents some limitations. First, although in the BRB-N the executive functions are underrepresented, due to time constraints we did not administer adjunctive tests of executive functions. Thus, it is not possible to completely rule out the possibility that patients presenting with a mild degree of executive dysfunction may have not been identified as ‘cognitively impaired’ by our neuropsychological assessment. In addition, we did not investigate the construct of cognitive reserve, that has been recently proposed as a possible ‘mediating’ factor for cognitive deficits in MS.

To conclude, our study corroborated the evidence that cognitive deficits are a common and important clinical feature of MS that should be carefully investigated at the early stages of the disease. As cognitive assessment has not yet gained the status of routine clinical examination, we strongly support the view that a neuropsychological screening assessment should be planned as part of the standard neurological examination of patients, especially when the demographic and clinical factors of the patient at hand (such as the type of MS and its duration, the level of disability status, and his/her pre-morbid level of verbal competence) suggest that cognitive impairment may be present. Furthermore, research should put more efforts in boosting the essential issue of cognitive rehabilitation. Regarding this, both medication and cognitive rehabilitation options need to be considered. Data are limited about the significant and stable effects of immune-modulating agents on cognitive impairment. However, clinical trials have suggested that such disease modifying therapies improve some aspects of the cognitive domain [30]–[31]. Another possible therapeutic option for managing cognitive disorders in MS encompasses the use of medications for clinical symptoms. For example, recent studies suggest that psycho-stimulants present an opportunity for adjunctive symptomatic therapy for slowed information processing and deficits of attention in MS, even if the their results need to be replicated in larger samples of patients [32]–[33]. Another promising therapeutic option is represented by cognitive rehabilitation. Cognitive rehabilitation in MS is still in its infancy. As noted in authoritative reviews of this emerging literature [34]–[35], the current findings about the efficacy of cognitive rehabilitation are mixed, with some studies showing encouraging results while others do not.

In conclusion, to date the best management approach appears to be to investigate the probable presence of cognitive impairment early during the course of the disease, and to provide timely and appropriate support for patients and families in order to minimize the psychological, social and professional impact of cognitive impairment in their lives. However, at this point there is no definitive treatment for cognitive deficits in MS. In addition, due to the fluctuation of cognitive symptoms amongst patients and during the course of the disease, it will be probably a good choice to take into due account individual patient's needs and preferences when planning cognitive interventions, instead of trying to apply standard protocols to all patients.
